# Supplementary material for: High-phytate/low-calcium diet is a risk factor for crystal nephropathies, renal phosphate wasting, and bone loss
Source: eLife. 2020 Apr 9;9:e52709. doi: 10.7554/eLife.52709 (PMC7145417; doi:10.7554/eLife.52709)
Supplement: Supplementary file 1. [file elife-52709-supp1.docx]

**High-phytate/low-calcium diet is a risk factor for crystal nephropathies, renal phosphate wasting, and bone loss**

**Supplementary File 1**. Composition of the AIN-93G rodent diets for experimental studies with rats

| AIN-93G Purified Rodent Diet | Control | HP-LCa^2+^ diets | | | HP-HCa^2+^ diets |
| --- | --- | --- | --- | --- | --- |
| Phytate (%) | 0 | 1 | 3 | 5 | 3 |
| Ingredient | (g/kg) | | | | |
| Casein | 200.00 | 200.00 | 200.00 | 200.00 | 200.00 |
| Cornstarch | 397.49 | 397.49 | 397.49 | 397.49 | 397.49 |
| Dyetrose | 132.00 | 132.00 | 132.00 | 132.00 | 132.00 |
| Cellulose | 50.00 | 50.00 | 50.00 | 50.00 | 50.00 |
| Soybean Oil | 70.00 | 70.00 | 70.00 | 70.00 | 70.00 |
| t-Butylhydroquinone | 0.01 | 0.01 | 0.01 | 0.01 | 0.01 |
| Salt Mix without Ca and Pi | 28.44 | 28.44 | 28.44 | 28.44 | 28.44 |
| Vitamin Mix | 10.00 | 10.00 | 10.00 | 10.00 | 10.00 |
| L-Cystine | 3.00 | 3.00 | 3.00 | 3.00 | 3.00 |
| Choline Bitartrate | 2.50 | 2.50 | 2.50 | 2.50 | 2.50 |
| Sucrose | 100.00 | 90.00 | 70.00 | 50.00 | 65.00 |
| Phytate* | 0.00 | 10.00 | 30.00 | 50.00 | 30.00 |
| *Phosphate in Phytate | 0.00 | 2.82 | 8.46 | 14.10 | 8.46 |
| Inorganic Phosphate | 1.56 | 1.56 | 1.56 | 1.56 | 1.56 |
| Ca | 5.00 | 5.00 | 5.00 | 5.00 | 10.00 |

| Mineral Mix (use at 28.44 g/kg diet), mg/kg |  |  | Vitamin Mixture (use at 10 g/kg Diet), mg/kg | | | | |  |
| --- | --- | --- | --- | --- | --- | --- | --- | --- |
| K | 3,600.00 |  | Niacin | | | | 3,000 |  |
| Na | 1,019.00 |  | Calcium Pantothenate | | | | 1,600 |  |
| Cl | 1,571.00 |  | Pyridoxine HCl | | | | 700 |  |
| S | 300.00 |  | Thiamine HCl | | | | 600 |  |
| Mg | 507.00 |  | Riboflavin | | | | 600 |  |
| Fe | 35.00 |  | Folic Acid | | | | 200 |  |
| Cu | 6.00 |  | Biotin | | | | 20 |  |
| Mn | 10.00 |  | Vitamin E Acetate (500 IU/g) | | | | 15,000 |  |
| Zn | 30.00 |  | Vitamin B12 (0.1%) | | | | 2,500 |  |
| Cr | 1.00 |  | Vitamin A Palmitate (500,000 IU/g) | | | | 800 |  |
| I | 0.20 |  | Vitamin D3 (400,000 IU/g) | | | | 250 |  |
| Se | 0.15 |  | Vitamin K1/Dextrose Mix (10 mg/g) | | | | 2,500 |  |
| F | 1.00 |  | Sucrose | | | | 967,230 |  |
| B | 0.50 |  |  |  |  |  |  | |
| Mo | 0.15 |  |  |  |  |  |  | |
| Si | 5.00 |  |  |  |  |  |  | |
| Ni | 0.50 |  |  |  |  |  |  | |
| Li | 0.10 |  |  |  |  |  |  | |
| V | 0.10 |  |  |  |  |  |  | |
