# Supplementary material for: High-phytate/low-calcium diet is a risk factor for crystal nephropathies, renal phosphate wasting, and bone loss
Source: eLife. 2020 Apr 9;9:e52709. doi: 10.7554/eLife.52709 (PMC7145417; doi:10.7554/eLife.52709)
Supplement: Supplementary file 2. [file elife-52709-supp2.docx]

**High-phytate/low-calcium diet is a risk factor for crystal nephropathies, renal phosphate wasting, and bone loss**

**Supplement File 2**. The nutrient compositions for three different diets.

|  | Wite rice (WR) | Brown rice (BR) | Brown rice + Rice bran (BR+RB) |
| --- | --- | --- | --- |
| Energy (kcal) | 2,280.8 ± 176.2 | 3,219.5 ± 176.2 | 3,818.6 ± 165.4 |
| Carbohydrates (g) | 369.0 ± 46.3 | 574.2 ± 46.3 | 540.9 ± 45.2 |
| lipids (g) | 49.7 ± 8.6 | 59.6 ± 8.6 | 112.9 ± 8.5 |
| Protein (g) | 93.5 ± 10.0 | 118.0 ± 0.0 | 151.7 ± 9.3 |
| Fibers (g) | 48.4 ± 11.4 | 61.5 ± 11.4 | 82.0 ± 10.6 |
| Moisture (water) | 1,165.1 ± 136.2 | 1,188.4 ± 136.2 | 1,195.0 ± 175.5 |
| Vitamin A (ug RE) | 698.7 ± 283.2 | 698.0 ± 283.2 | 691.6 ± 285.1 |
| Vitamin D (ug) | 3.8 ± 4.3 | 3.8 ±4.3 | 3.8 ± 4.3 |
| Vitamin E (mg) | 37.1 ± 9.8 | 42.3 ± 9.8 | 41.1 ± 9.4 |
| Vitamin K (ug) | 310.3 ± 109.4 | 310.3 ± 109.4 | 310.3 ± 109.4 |
| Vitamin C (mg) | 200.2 ± 25.2 | 200.2 ±25.2 | 98.2 ± 28.5 |
| Thiamine (mg) | 2.1 ± 0.4 | 2.9 ± 0.4 | 10.3 ± 0.4 |
| Riboflavin (mg) | 2.1 ± 0.6 | 2.4 ± 0.6 | 3.8 ± 0.6 |
| Niacin (mg) | 21.6 ± 4.7 | 36.9 ± 4.7 | 109.2 ± 4.6 |
| Vitamin B6 (mg) | 3.0 ± 0.8 | 4.8 ± 0.8 | 4.5 ± 0.8 |
| Folic acid (ug) | 1,091.8 ± 131.8 | 1,157.5 ± 131.8 | 1,108.3 ± 129.0 |
| Vatamin B12 (ug) | 10.6 ± 1.6 | 10.6 ± 1.6 | 10.6 ± 1.6 |
| Vitamin B5 (ug) | 5.3 ± 1.1 | 9.8 ±1.1 | 8.7 ± 1.1 |
| Biotin (ug) | 2.3 ± 2.7 | 2.3 ± 2.7 | 2.3 ± 2.7 |
| Ca (mg) | 966.6 ± 231.8 | 982.8 ± 231.8 | 1,098.9 ± 197.5 |
| Pi (mg) | 1,517.6 ± 84.6 | 2,695.1 ± 84.6 | 7,283.5 ± 67.6 |
| Na (mg) | 6,470.3 ± 674.9 | 6,839.0 ± 674.9 | 6,718.4 ± 623.4 |
| Cl (mg) | 33.0 ± 11.5 | 33.0 ± 11.5 | 33.0 ± 11.5 |
| K (mg) | 5,126.5 ± 1,372.0 | 6,498.1 ± 1,372.0 | 11,609.5 ± 1,302.3 |
| Mg (mg) | 148.1 ± 18.4 | 148.1 ± 18.4 | 3,148.1 ± 18.4 |
| Fe (mg) | 30.4 ± 5.3 | 30.8 ± 5.3 | 48.1 ± 5.2 |
| Zn (mg) | 13.1 ± 1.8 | 18.6 ± 1.8 | 35.8 ± 1.6 |
| Cu (ug) | 926.4 ± 231.8 | 2,256.0 ± 0.2 | 3,916.5 ± 231.8 |
| F (ug) | 0.1 ± 0.1 | 1.0 ± 0.1 | 0.1 ± 0.1 |
| Mn (mg) | 3.9 ± 2.1 | 8.3 ± 2.1 | 7.6 ± 2.1 |
| I (ug) | 533.2 ± 766.8 | 533.2 ± 766.8 | 533.2 ± 766.8 |
| Se (ug) | 80.1 ± 14.1 | 192.4 ± 14.1 | 175.6 ± 14.1 |
| Co (ug) | 0.3 ± 0.6 | 0.3 ± 0.6 | 0.3 ± 0.6 |
| Mo (ug) | 2.5 ± 0.7 | 2.5 ± 0.7 | 2.5 ± 0.7 |
