# Supplementary material for: High-phytate/low-calcium diet is a risk factor for crystal nephropathies, renal phosphate wasting, and bone loss
Source: eLife. 2020 Apr 9;9:e52709. doi: 10.7554/eLife.52709 (PMC7145417; doi:10.7554/eLife.52709)
Supplement: Supplementary file 3. [file elife-52709-supp3.docx]

**High-phytate/low-calcium diet is a risk factor for crystal nephropathies, renal phosphate wasting, and bone loss**

**Supplement File 3**. Biochemical properties during three consecutive diets.

| repeated measure ANOVA | | White rice | Brown rice | Brown rice +  Rice bran | | between Subjects | | between Group |
| --- | --- | --- | --- | --- | --- | --- | --- | --- |
|  | | (n = 6) | (n = 6) | (n = 6) | | P value | | P value |
| BUN (mg/dL) | 11.10 ± 2.54 | | 10.13 ± 1.52 | | 9.53 ± 1.62 | | 0.0012 | 0.0534 |
| Cr (mg/dL) | 0.59 ± 0.09 | | 0.59 ± 0.08 | | 0.61 ± 0.05 | | 0.0001 | 0.2767 |
| Glucose (mg/dL) | 89.17 ± 6.27 | | 78.50 ± 4.14 | | 81.83 ± 5.23 | | 0.1573 | 0.0067 |
| T.Chol (mg/dL) | 156.33 ± 29.49 | | 143.33 ± 28.02 | | 142.00 ± 26.53 | | <0.0001 | 0.0002 |
| LDL (mg/dL) | 94.50 ± 23.92 | | 85.83 ± 23.89 | | 81.17 ± 22.33 | | <0.0001 | 0.0022 |
| TG (mg/dL) | 70.00 ± 15.75 | | 56.83 ± 13.67 | | 65.33 ± 26.87 | | 0.0374 | 0.3110 |
| HDL(mg/dL) | 56.00 ± 6.23 | | 54.83 ± 9.11 | | 54.83 ± 10.82 | | 0.0001 | 0.8056 |

Statistical significance were tested by repeated measure ANOVA between subjects and groups. Data are mean ± SD. T.Cho, total cholesterol, LDL, low density lipoprotein, TG, triglyceride, HDL, high density lipoprotein.
