# Supplementary material for: High-phytate/low-calcium diet is a risk factor for crystal nephropathies, renal phosphate wasting, and bone loss
Source: eLife. 2020 Apr 9;9:e52709. doi: 10.7554/eLife.52709 (PMC7145417; doi:10.7554/eLife.52709)
Supplement: Supplementary file 4. [file elife-52709-supp4.docx]

**High-phytate/low-calcium diet is a risk factor for crystal nephropathies, renal phosphate wasting, and bone loss**

**Supplement File 4. Phylum- and class-specific primers for real-time PCR**

| Gene | | Primer sequence | |
| --- | --- | --- | --- |
| *Eubacteria* | Forward: 5’-AAA CTC AAA KGA ATT GAC GG -3’  Reverse: 5’-GCT GCC TCC CGT AGG AGT-3’ | |  |
| *Bacteroidetes* | Forward: 5’-GGT TCT GAG AGG AAG GTC CC-3’  Reverse: 5’-TTA ASC CGA CAC CTC ACG G-3’ | |  |
| *Firmicutes* | Forward: 5’-GGA GYA TGT GGT TTA ATT CGA AGC A -3’  Reverse: 5’-AGC TGA CGA CAA CCA TGC AC-3’ | |  |
| *Actinobacteria* | Forward: 5’-TGT AGC GGT GGA ATG CGC-3’  Reverse: 5’-AAT TAA GCC ACA TGC TCC GCT-3’ | |  |
| *Betaproteobacteria* | Forward: 5’-AAC GCG AAA AAC CTT ACC TAC C-3’  Reverse: 5’-TGC CCT TTC GTA GCA ACT AGT G-3’ | |  |
