# Supplementary material for: High-phytate/low-calcium diet is a risk factor for crystal nephropathies, renal phosphate wasting, and bone loss
Source: eLife. 2020 Apr 9;9:e52709. doi: 10.7554/eLife.52709 (PMC7145417; doi:10.7554/eLife.52709)
Supplement: Supplementary file 5. [file elife-52709-supp5.docx]

**High-phytate/low-calcium diet is a risk factor for crystal nephropathies, renal phosphate wasting, and bone loss**

**Supplement File 5**. Biochemical and structural properties of mammalin, gut microbial, and bacterial phytases.

| Source | | Phylum | Class | Optimum pH | Active site | Crystal structure | Substrate | Effects of Ca^2+^ | Effects of EDTA | Signal sequences | ER retention signal (KDEL) |
| --- | --- | --- | --- | --- | --- | --- | --- | --- | --- | --- | --- |
| Mammalian | |  | MINPP-1 | 2.0-5.0 | Basic (+) | α/β | Ca^2+^-free phytate | - | + | Yes | Yes |
| Bacteria | Anaerobic | Firmicutes | None | None | None | None | None | None | None | None | None |
|  |  | Bacteriodetes | HAP | 2.0-5.0 | Basic (+) | α/β | Ca^2+^-free phytate | - | + | Yes | No |
|  |  | βProteobacter | HAP | 2.0-5.0 | Basic (+) | α/β | Ca^2+^-free phytate | - | + | Yes | No |
|  |  | Actinobacteria | HAP | 2.0-5.0 | Basic (+) | α/β | Ca^2+^-free phytate | - | + | Yes | No |
|  | Aerobic | Firmicutes | BPP | 5.0-8.0 | Acidic (-) | β-propeller | Ca^2+^-phytate | + | - | Yes | No |
|  |  | Bacteriodetes | BPP | 5.0-8.0 | Acidic (-) | β-propeller | Ca^2+^-phytate | + | - | Yes | No |
|  |  | βProteobacter | BPP | 5.0-8.0 | Acidic (-) | β-propeller | Ca^2+^-phytate | + | - | Yes | No |
|  |  | Actinobacteria | BPP | 5.0-8.0 | Acidic (-) | β-propeller | Ca^2+^-phytate | + | - | Yes | No |

MINPP1, mammalian inositol polyphosphate phosphatase, HAP, histidine acid phosphatase, BPP, β-propeller phytase
