# Supplementary material for: High-phytate/low-calcium diet is a risk factor for crystal nephropathies, renal phosphate wasting, and bone loss
Source: eLife. 2020 Apr 9;9:e52709. doi: 10.7554/eLife.52709 (PMC7145417; doi:10.7554/eLife.52709)
Supplement: Supplementary file 6. [file elife-52709-supp6.docx]

**High-phytate/low-calcium diet is a risk factor for crystal nephropathies, renal phosphate wasting, and bone loss**

**Supplement File 6**. The qRT-PCR primer lists

|  | Gene | GenBank accession no. | Primer | Primer sequences | Amplicon-size, bp | |
| --- | --- | --- | --- | --- | --- | --- |
| 1 | CYP27B1 | NM_053763.1 | Forward | 5'-TCCCAGCTACCCCTGCTAAAG | 85 | |
|  |  |  | Reverse | 5'-CTGTCTGGGACACGGGAGTT |  |  |
| 2 | CYP24A1 | NM_201635.2 | Forward | 5'-GAACGAAGCCTACGGGTTGA | 91 | |
|  |  |  | Reverse | 5'-CTCCACGGGCTTCATGAGTT |  |  |
| 3 | VDR | NM_017058.1 | Forward | 5'-TCCCAGGATTCAGGGATCTCA | 96 | |
|  |  |  | Reverse | 5'-TGAAAGACTGGTTGGAGCGT |  |  |
| 4 | CLCN5 | NM_017106.1 | Forward | 5'-TATTACCACCACGACTGGGG | | 119 |
|  |  |  | Reverse | 5'-ATCCGAGTCACACCGCCTAA | |  |
| 5 | ENPP1 | NM_053535.1 | Forward | 5'-GAGCTTCCTGTCCCAGTGTC | | 80 |
|  |  |  | Reverse | 5'-TGATCGGCACAATCGAAGGG | |  |
| 6 | OCRL | NM_001108256.2 | Forward | 5'-TCCTGAGACAATCCCTGGTAGT | | 116 |
|  |  |  | Reverse | 5'-CAGAGTCGAGACATCGCTGA | |  |
| 7 | LRP5 | NM_001106321.2 | Forward | 5'-ATGGCAAGACGTGTAAGGCA | | 86 |
|  |  |  | Reverse | 5'-AGGGGTGTCCAGAGAGATCC | |  |
| 8 | NHERF1 | NM_021594.1 | Forward | 5'-ACAGAAGGAGAACAGCCGTG | | 96 |
|  |  |  | Reverse | 5'-TCCTCACTGGTATCGCTGGA | |  |
| 9 | PHEX | NM_013004.1 | Forward | 5'-ACTGCAAACACTGGCAACATT | | 119 |
|  |  |  | Reverse | 5'-GTGTTGCTTGGTCCAACTTCAA | |  |
| 10 | ITPR1 | NM_001007235.2 | Forward | 5'-GCGTGGCTCCCCGGT | | 116 |
|  |  |  | Reverse | 5'-TCAGACATGTCCTTGTTGAAAAGC | |  |
| 11 | CasR | NM_016996.1 | Forward | 5'-GGATTTGGAGGCTGGAGACCC | | 120 |
|  |  |  | Reverse | 5'-CCTTGGGTCAAGCTACTGCCT | |  |
| 12 | NaPi-2a | NM_013030.1 | Forward | 5'-CTCTGCTGAAGGGCCAAGTG | | 82 |
|  |  |  | Reverse | 5'-CCTGTGACCCAAGTGAAGGG | |  |
| 13 | αKlotho | NM_031336.1 | Forward | 5'-CATGCCGAGCAAGACTCACT | | 94 |
|  |  |  | Reverse | 5'-CACAAAGGTTGATGCCGTCC | |  |
| 14 | NGAL | NM_130741.1 | Forward | 5'-ACGTCACTTCCATCCTCGTC | | 97 |
|  |  |  | Reverse | 5'-ATATTCCCCAGGGTGAACTGG | |  |
| 15 | COL1A1 | NM_053304.1 | Forward | 5'-TGACGCATGGCCAAGAAGAC | | 84 |
|  |  |  | Reverse | 5'-CAGGTTTCCACGTCTCACCAT | |  |
| 16 | COL6A2 | NM_001100741.1 | Forward | 5'-CATGGAAGACGTCCTTTGTCC | | 85 |
|  |  |  | Reverse | 5'-TGTGCACTGGGCCACATAGA | |  |
| 17 | MPG | NM_012862.1 | Forward | 5'-ACACCCGAGACCATGAAGAGC | | 119 |
|  |  |  | Reverse | 5'-GTGGTGAAGGGACTGACTTCATA | |  |
